# Bacteria and Fermentation

**Published:** 1877-04

**Authors:** 


### ARTICLE VII.

### *Bacteria and Fermentation.*

A granular powder is placed in your hands, and you are asked to state what it is. You examine it, and have or have not reason to suspect that seeds of some kind are mixed up in it. But you prepare a bed in your garden, sow in it the powder, and soon after find a mixed crop of docks and thistle sprouting from your bed. Until this powder was sown neither docks or thistles ever made their appearance in your garden. You repeat the experiment once, twice, ten times, fifty times. From fifty different beds, after the sowing of the powder, you obtain the same crop. What will be your response to the question proposed to you ? "I am not in a condition," you would say, "to affirm that every grain of the powder is a dock-seed or a thistle-seed, but I am in a condition to affirm that both dock and thistle-seeds form at all events, part of the powder." Supposing a succession of such powders to be placed in your hands, with grains becoming gradually smaller until they dwindled to the size of impalpable dust particles; assuming that you treat them all in the same way, and that from every one of them in a few days you obtain a definite crop—it may be clover, it may be mustard, it may be mignonette, it may be a plant more minute than any of these—the smallness of the particles, or of the plants that spring from them, does not affect the validity of the conclusion—without a shadow of misgiving you would conclude that the powder must have contained seeds or germs of the life observed. There is not in the range of physical science an experiment more conclusive, nor an inference safer than this one. Supposing the powder to be light enough to float in the air, and that you are enabled to see it there just as plainly as you saw the heavier powder in the palm of your hand—if the dust sown by the air instead of by the hand produce a definite living crop—with some logical rigor you would conclude that the germs of this crop must be mixed with the dust. To take an illustration, the spores of the listle plant *Penicillium glancum*, to which I have already referred, are light enough to float in the air. A cut apple, a pear, a tomato, a slice of vegetable marrow, or, as already mentioned, an old moist boot, a dish of paste, or a pot of jam, constitutes a proper soil for the *Penicillium.* Now, if it could be proved that the dust of the air, when sown in this soil, produces this plant—while, wanting the dust, neither the air, nor the soil, nor both together can produce it—it would be obviously just as certain in this case that the floating dust contains the germ of *Penicillium* as that the powders sown in your garden contained the germs of the plants which sprung from them. But how is the floating dust to be rendered visible? In this way. Build a little chamber and provide it with a door, window and window-shutters. Let an aperture be made in one of the shutters, through which a sunbeam can pass. Close the door and windows so that no light shall enter save through the hole in the shutter. The track of the sunbeam is at first perfectly plain and vivid in the air of the room. If all disturbance of the air of the chamber be avoided, the luminous track will become fainter and fainter, until at last it disappears absolutely, and no trace of the beam is to be seen. What rendered the beams visible at first? The floating dust of the air, which thus illuminated and observed, is as palpable to sense as any dust or powder placed on the palm of the hand. In the still air the dust gradually sinks to the floor or sticks to the walls or ceiling, until finally, by this self-cleansing process, the air is entirely freed from mechanically suspended matter. Thus far I think we have made our footing sure. Let us proceed. Chop up a beefsteak, and allow it to remain for two or three hours just covered with warm water; you thus extract the juice of the beef in a concentrated form. By properly boiling the liquid and filtering it, you can obtain from it a perfectly transparent beef tea. Expose a number of vessels containing this tea to the moteless air of your chamber, and expose a number of similar vessels containing precisely the same liquid to the dust-laden air; in three days every one of the latter stinks, and, examined with the microscope, every one of them is swarming with the bacteria of putrefaction. After three months or three years the beef-tea within the chamber is found, in every case, as sweet and clear, and as free from bacteria, as it was at the moment when it was first put in. There is absolutely no difference between the air within and that without, save that the one is dustless and the other dust-laden. Clinch the experiment thus: Open the door of your chamber and allow the dust to enter it; in three days afterward you have every vessel within the chamber swarming with bacteria and in a state of active putrefaction. Here, also, the inference is quite as certain in the case of the powder sown in your garden. Multiply your proofs by building fifty chambers instead of one, and by employing every imaginable infusion of wild animals and tame; of flesh, fish, fowl, and viscera; of vegetable of the most various kinds. If in all these cases you find the dust infallibly producing its crop of bacteria, while neither the dustless air, nor the nutritive infusion, nor both together, are ever able to produce this crop, your conclusion is simply irresistible that the dust of the air contains the germs of the crops which has appeared in your infusions. I repeat, there is no inference of experimental science more certain than this one. In the presence of such facts, to use the words of a paper lately published in the "Philosophical Transactions," it would be simply monstrous to affirm that these swarming crops of bacteria are spontaneously generated. Is there then no experimental proof of spontaneous generation? I answer without hesitation—none. But, to doubt the experimental proof of a fact, and to deny its possibility, are two different things, though some writers confuse matters by making them synonymous. In fact, this doctrine of spontaneous generation, in one form or another, falls in with the theoretic beliefs of some of the foremost workers of this age; but it is exactly these men who have the penetration to see and the honesty to expose the weakness of the evidence adduced in its support.—*Drug. Circular, from a Lecture by Prof. Tyndall.*

